# Ameliorating effect of probiotic on nonalcoholic fatty liver disease and lipolytic gene expression in rabbits

**DOI:** 10.1038/s41598-023-32584-7

**Published:** 2023-04-18

**Authors:** Marina Aziz, Shabaan A. Hemeda, Ghadeer M. Albadrani, Sabreen E. Fadl, Fatma Elgendey

**Affiliations:** 1 Genetics and Genetic Engineering, Department of Animal Wealth Development, Faculty of Veterinary Medicine, Benha University, Banha, Egypt; 2 Department of Animal Husbandry and Animal Wealth Development, Faculty of Veterinary Medicine, Alexandria University, Alexandria, Egypt; 3 Department of Biology, College of Science, Princess Nourah bint Abdulrahman University, P.O. Box 84428, Riyadh, 11671 Saudi Arabia; 4 Biochemistry Department, Faculty of Veterinary Medicine, Matrouh University, Matrouh, Egypt

**Keywords:** Biochemistry, Molecular biology, Physiology

## Abstract

Nonalcoholic fatty liver disease (NAFLD) is a condition that affects about 24% of people worldwide. Increased liver fat, inflammation, and, in the most severe cases, cell death are all characteristics of NAFLD. However, NAFLD pathogenesis and therapy are still not clear enough. Thus, this study aimed to determine the effect of a high-cholesterol diet (HCD) inducing NAFLD on lipolytic gene expression, liver function, lipid profile, and antioxidant enzymes in rabbits and the modulatory effects of probiotic *Lactobacillus acidophilus* (*L*.* acidophilus)* on it. A total of 45 male New Zealand white rabbits, eight weeks old, were randomly divided into three groups of three replicates (5 rabbits/replicate). Rabbits in group I were given a basal diet; rabbits in group II were given a high-cholesterol diet that caused NAFLD; and rabbits in group III were given a high-cholesterol diet as well as probiotics in water for 8 weeks. The results showed that a high-cholesterol diet caused hepatic vacuolation and upregulated the genes for lipoprotein lipase (LPL), hepatic lipase (HL), and cholesteryl ester transfer protein (CETP). Downregulated low-density lipoprotein receptor (LDLr) gene, increased liver enzymes [alanine transaminase (ALT), aspartate transaminase (AST), alkaline phosphatase (ALP), lactate dehydrogenase (LDH)], cholesterol, triglycerides (TG), low-density lipoprotein (LDL), glucose, and total bilirubin. On the other hand, it decreased high-density lipoprotein (HDL), total protein, albumin, and liver antioxidants [glutathione peroxidase (GPx), catalase (CAT), reduced glutathione (GSH), and superoxide dismutase (SOD)]. Supplementing with probiotics helped to return all parameters to normal levels. In conclusion**,** probiotic supplementation, especially *L. acidophilus,* protected against NAFLD, and restored lipolytic gene expression, liver functions, and antioxidants to normal levels.

## Introduction

The percentage of nonalcoholic fatty liver disease (NAFLD) patients who also have nonalcoholic steatohepatitis (NASH) is expected to increase during the coming ten years. One modelling study predicts that by 2030, there will be an increase in NAFLD prevalence of 18%. There will be 27 million NASH patients in the US, a 56 percent increase from the current number^1^. NAFLD is associated with an increased chance of passing away, particularly from heart disease, hepatocellular cancer, and liver-related incidents^2^. Moreover, NAFLD is consistently seen in bladder cancer patients^3^. According to the increase in frequency in recent decades, it has emerged as the second most common reason for liver transplantation in the United States^4^. Hepatic steatosis, NASH, liver cirrhosis, and hepatocellular cancer are all considered to be part of the nonalcoholic fatty liver disease group of liver illnesses^5^.

The development and progression of NAFLD have been linked to a number of genes. NAFLD has been most strongly associated with the single nucleotide polymorphism (SNP) causing isoleucine to methionine substitution at position 148 in the patatin-like phospholipase domain-containing 3 (PNPLA3). Triacylglycerol, diacylglycerol, and monoacylglycerol are hydrolyzed by PNPLA3, but the I148M mutation renders the enzyme inactive^6^. This genetic variant is associated with increased NASH activity, increased liver lipid content, and an increased chance of developing hepatocellular cancer and liver fibrosis^7,8^. Three more well-researched genetic variants include glucokinase regulator (GCKR) P446L, membrane-bound O-acyltransferase domain-containing 7 (MBOAT7), and transmembrane 6 superfamily member 2 (TM6SF2), rs58542926 C > T. They also raise the risk of fibrosis and increase the severity of NAFLD^9,10^.

The lipase gene family includes lipoprotein lipase (LPL), pancreatic lipase, hepatic lipase, and endothelial lipase. The gene converts lipoprotein triglycerides into one monoacylglycerol molecule and two free fatty acids. Chylomicrons and very low density lipoprotein (VLDL) contain it. Additionally, it facilitates the uptake of free fatty acids, cholesterol-rich lipoproteins, and leftover chylomicrons into cells^11^. The LPL gene encodes lipoprotein lipase, which is expressed in the heart, muscles, and adipose tissue^12^. Low-density lipoprotein receptors are crucial for regulating blood cholesterol levels. They are particularly prevalent in the liver, which is capable of getting rid of the majority of the extra cholesterol in the body. The number of low density lipoprotein receptors (LDLr) on the surface of liver cells controls how rapidly cholesterol is removed from the bloodstream^13–15^. The LDLr gene has 18 exons and is located in band 19p13.2 on chromosome 19^16^. The hydrolysis of triacylglycerides is catalyzed by hepatic lipase (HL). Other names for it include hepatic triglyceride lipase (HTGL)^17^. Chromosome 15 contains the HL gene^18^. Hepatic lipase is mostly expressed in hepatocytes and endothelial cells in the liver. There are two possibilities for hepatic lipase: it can either stay attached to the liver or it can separate from liver endothelial cells and be free to enter the bloodstream^19^. Cholesteryl ester transfer protein gene (CETP), a protein that reduces high density lipoprotein (HDL) levels by transferring cholesteryl esters from HDL to particles that contain apolipoprotein B in exchange for triglycerides. Triglycerides and cholesterol esters are transported between VLDL, LDL, and HDL via the enzyme CETP. Lower CETP levels promote the synthesis of HDL. Since higher HDL levels are associated with a decreased risk of atherosclerosis^20^. The genomic DNA for the CETP gene has about 25 Kbp and 16 exons^21^. The upregulation of CETP is brought on by either an increase in dietary cholesterol or endogenous hypercholesterolemia^22^.

In numerous studies, probiotic treatment has been found to decrease the symptoms of NAFLD in animal models^23–25^. Probiotics improve liver function, restore the gut flora, and improve the lipid profile by assessing circulating total cholesterol, HDL, LDL, and triglycerides^25^. One of the most prevalent genera of probiotic bacteria, *Lactobacillus*, has a long history of safe use and is recognized by the US Food and Drug Administration as safe for human consumption^26^. The researchers found that the strain most successful at reducing total cholesterol (TC) and low-density lipoprotein cholesterol (LDL-C) was *Lactobacillus acidophilus*^27,28^. The most popular probiotics, *Lactobacillus* species, are given for a lengthy period^29^. It was discovered to prevent NAFLD brought on by a high-fat diet (HFD) and to enhance gut permeability, inflammation, and gut flora modulation in a diet-induced obesity model^30–32^. The interaction of probiotic bacteria with bile acids via deconjugation events catalyzed by bile salt hydrolase enzymes (BSH) is assumed to be the basic mechanism of their cholesterol-lowering properties^33^. Probiotic strains containing BSH help deconjugate bile salts, which is the first stage in the colon's biotransformation of bile salts. Deconjugation is the term for the enzymatic dissolution of the C-24 N-acyl amide link connecting bile acids to their amino acid conjugates^34^.

So, this study aimed to determine the effect of a high cholesterol diet (HCD) inducing NAFLD on lipolytic gene expression, liver function, lipid profile, and antioxidant enzymes in rabbits and the modulatory effects of probiotic *Lactobacillus acidophilus* on it.

## Material and methods

The current study was approved by the Ethical Committee for live birds sampling at the Faculty of Veterinary Medicine, Benha University (BUFVTM 01-09-22).

### Rabbits

The current study was carried out in the Faculty of Veterinary Medicine, Benha University, Department of Animal Wealth Development, using 45 male New Zealand white rabbits (eight weeks old, about 1200 g body weight). They were obtained from SAN El-HAGAR, ASH SHARQIYAH, EGYPT. All animal handling procedures are in agreement with the ARRIVE guidelines from the National Center for the Replacement, Refinement, and Reduction of Animals in Research (NC3Rs) ^19^ throughout the experimental period (eight weeks).

### The experimental design

The rabbits were weighed individually and marked. The rabbits were divided into three groups at random; each group had three replicates of five rabbits. The rabbits were housed in wire mesh cages with identical housing and care practices; feed was applied twice a day, and water was applied constantly by the nipple system. The home was clean, disinfected, and well-ventilated, with the right environmental temperature. Lighting was provided for 16 h: 8 h of darkness throughout the experimental period. All methods were carried out in accordance with relevant guidelines and regulations.

The three groups were as follows:

- Group I received the basal diet.
- Group II received a high cholesterol diet (HCD), a 2% cholesterol diet^35^.
- Group III received HCD with probiotic (1 g/L water) in water. The probiotic *Lactobacillus acidophilus* (Lacto biotech^®^) is produced and exported by Mycrofeed Srl, Italy, and obtained by Cairomed Pharmaceuticals Company.

The experimental food was introduced to the rabbits gradually over a two-week adaptation period, the trial lasted for eight weeks. The ingredients and nutritional composition of the basal diet and HCD are represented in Tables 1 and 2.

**Table 1:** Ingredients and nutritional composition of basal diet.

Ingredients	Amount (kg/ton)	%	Nutrients chemical composition		
			Component	Value	Unit
Berseem hay 16%	303.00	30.30	Digested energy	2600.92	Kcal/kg
Wheat bran	250.00	25.00	Crude protein	17.99	%
Soybean meal 46	175.00	17.50	Crude fiber	13.48	%
Yellow corn	136.00	13.60	Lysine	0.97	%
Fennel hay	50.00	5.00	Methionine + cystine	0.60	%
Molasses	30.00	3.00	Calcium	1.10	%
Glutafeed	27.00	2.70	Total phosphorus	0.70	%
Limestone	10.60	1.06	Chloride	0.23	%
Monosodium phosphate	8.25	0.83	Sodium	0.20	%
Salt	3.50	0.35			
Vitamin, mineral premix	3.00	0.30			
Bi sodium carbonate	1.90	0.19			
Anticoccidial	1.00	0.10			
Anti-mycotoxin	0.50	0.05			
D-L methionine	0.25	0.03

**Table 2:** Ingredients and nutritional composition of HCD.

Ingredients	Amount (Kg/ton)	%	Nutrients chemical composition		
			Component	Value	Unit
Berseem hay 16%	259.00	25.90	Digested energy	2,602.69	Kcal / kg
Wheat bran	249.00	24.90	Crude protein	17.99	%
Rice kernel	181.00	18.10	Cholesterol	100	Cholesterol
Soybean meal 46	123.00	12.30	Crude fat	5.00	%
Full fat soya	55.00	5.50	Crude fiber	13.55	%
Fennel hay	50.00	5.00	Lysine	0.99	%
Molasses	30.00	3.00	Methionine + cystine	0.59	%
Barely	28,00	2.80	Calcium	1.09	%
Limestone	16.00	1.60	Total phosphorus	0.70	%
Salt	3.50	0.35	Chloride	0.23	%
Vitamin, mineral premix	3.00	0.30	Sodium	0.20	%
Bi sodium carbonate	1.50	0.15			
Anticoccidial	0.50	0.05			
Antimycotoxin	0.50	0.05			

### Histopathological examination of the liver

Liver samples from various areas of the liver were obtained for histological evaluation and investigation for NAFLD. Samples were stored in 10% buffered neutral formalin, and then exposed to microscopic examination in accordance with the method described by Davis^36^.

Scoring of hepatic steatosis was done according Nassir et al.^37^ in which grading was done on the basis of the percentage of fat within the hepatocytes: grade 0 (healthy, < 5%), grade 1 (mild, 5%-33%), grade 2 (moderate, 34%-66%), and grade 3 (severe, > 66%).

### Determination of lipolytic genes expression

#### Samples collection

On day 56 following the commencement of the trial (at the end of eight weeks), 12 representative rabbits (selected at random as three rabbits per replicate) had been sacrificed by overdose injection of pentobarbital sodium at 60 to 70 mg/kg live weight for sampling. Liver samples had been taken and kept at − 80 °C for subsequent study.

#### Quantitative real-time PCR analysis

Following the manufacturer's instructions, 50 mg of tissue was homogenized in a sterile collection tube with 750 µl of Trizol solution using a rotor Tissue Ruptor to extract the total RNA (Qiagen, GmbH, Germany). By measuring the absorbance in a nanodrop spectrophotometer (BMG Lab Tec. GmbH, Germany), the concentration and purity of RNA were assessed. The A260/A280 ratio of undiluted RNA is (1.8–2.0). The primers were created using NCBI Primer-BLAST Software, and their sequences are displayed in Table 3. Two µg of total RNA was reverse transcribed into cDNA using 2X Reverse Transcriptase Master Mix (Applied Biosystem, USA) following the manufacturer's instructions. The Applied Biosystems 7500 Fast Real-time PCR, USA, was used to quantify the mRNA. The quantitative PCR was conducted using the SYBER Green Master Mix in a 20 µL reaction mixture (TOPreal TM qPCR 2X PreMIX). The initial activation (3 min/95 °C), denaturation (3 s/95 °C), and annealing/extension (30 s/60 °C) were used to justify the cycling condition, and 40 cycles were used in total, according to^38^. The GAPDH gene served as the standard for all gene expression levels. Utilizing the 2^-ΔΔCt^ technique, gene expression has been compared and quantified^39^.

**Table 3:** Primers used for qRT-PCR.

Gene name	Primer sequence (5′-3′)	Expected product size	Accession number
*GAPDH*	F- GCCGCTTCTTCTCGTGCAG R- ATGGATCATTGATGGCGACAACAT	145	L23961
*LPL*	F- ACAAGAGAGAACCAGACTCCAAC R- TCAGACTTCCAGCAATGCCAG	216	ENSOCUT00000008235
*LDLr*	F- TGCACTCCATCTCCAGCATC R- TCTTCTCGCACCAGTTCACC	264	M11501
*HL*	F- CTACATCAGCGGAAAGCACA R- GAGCTCCAGGAAGTGACAGC	241	AF041202
*CETP*	F-AGCTCTTCACAAACTTCATCTCCTTC R- CTTGTGATGGGACTCCAGGTAGG	206	M27486

*GAPDH* refers to Glyceraldehyde 3-phosphate dehydrogenase, *LPL* refers to Lipoprotein lipase, *LDLr* refers to low-density lipoprotein receptor,* HL* refers to hepatic lipase, and *CETP* refer to Cholesteryl ester transfer protein.

### Biochemical analysis of blood

At the completion of the study period, blood samples were taken from each animal. After an overnight fast, blood samples were taken from the ear vein of the rabbits by means of a serum-separating blood collection tube and a vacuum gel tube with a clot activator. Samples were drawn into dry, clean test tubes and allowed to clot for half an hour at room temperature to separate the serum. Clear sera were separated by centrifugation at 3500 rpm for 15 min and then collected using automatic micropipettes in Eppendorf’s tubes. The serum was maintained at − 20 °C in a deep freezer to determine the following parameters: alanine transaminase (ALT), aspartate transaminase (AST), alkaline phosphatase (ALP), lactate dehydrogenase (LDH), cholesterol, triglyceride (TG), high-density lipoprotein (HDL), low-density lipoprotein (LDL), total protein, albumin, glucose, and total bilirubin.

### Determination of liver antioxidants

Liver antioxidants were determined in liver tissue homogenate according to methods adopted by Weissman^40^ for glutathione peroxidase (GPx), Aebi^41^ for catalase (CAT), Beutler et al.^42^ for reduced glutathione (GSH), and Nishikimi et al.^43^ for superoxide dismutase (SOD).

### Statistical analysis

The statistical program SPSS was used to analyze the data (version 21; SPSS Inc., Chicago, IL, USA). The results were mean ± SE, according to the independent sample T-test analysis, Significant statistically (*P* ≤ 0.05).

### Ethical approval

The current study was approved by the Ethical Committee for live birds sampling at the Faculty of Veterinary Medicine, Benha University (BUFVTM 01-09-22).

### Guidelines

All methods were carried out in accordance with relevant guidelines and regulations.

### ARRIVE guidelines

The authors confirm that the study was carried out in compliance with the ARRIVE guidelines.

## Result

**Figure 1:**
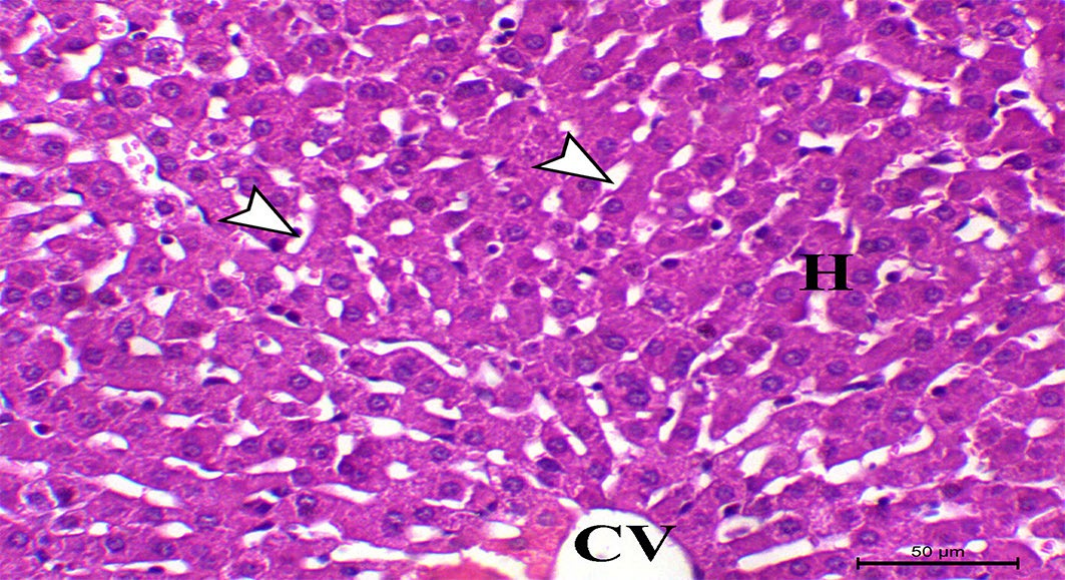
Photomicrograph of the hepatic section of control rabbit, showing normal hepatic cells (H letters) arranged in cords and searated with sinusoids (arrowheads) around the central vein (CV), (H&E stain), X200, bar = 50 µm.

**Figure 2:**
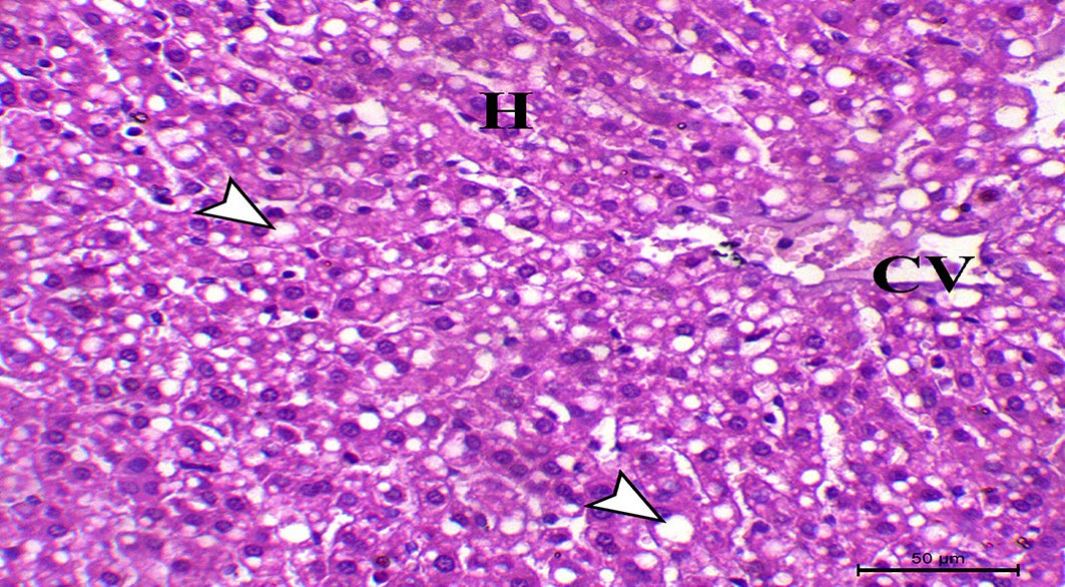
Photomicrograph of the hepatic section of HCD supplemented rabbit, showing marked hepatic vacuolation associated with fat cytoplasmic vacuoles (arrowheads) (CV indicates central vein and H indicates hepatocytes), (H&E stain), X200, bar = 50 µm.

**Figure 3:**
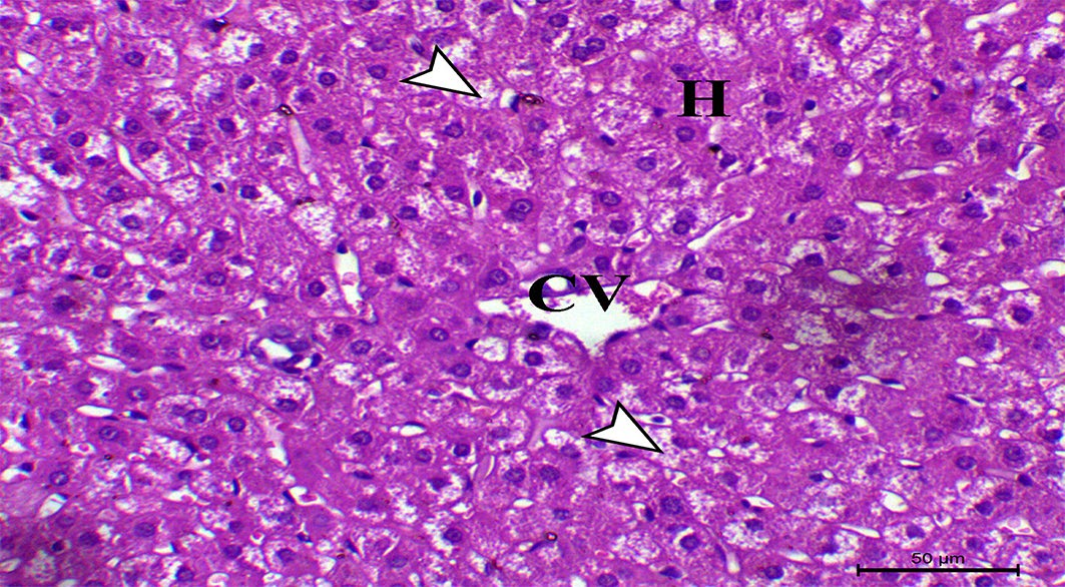
Photomicrograph of the hepatic section of HCD with probiotic supplemented rabbit showing marked decrease hepatic fatty changes with mild glycogen infiltration (arrowheads) (CV indicates central vein and H indicates hepatocytes), (H&E stain), X200, bar = 50 µm.

Concerning histopathological changes in liver sections from control rabbits, they showed the liver's typical histological structure, which was made up of hepatic lobules with radiating hepatocytes surrounding a central vein and irregular blood sinusoids separating them (Fig. 1). HCD supplemented group exhibited pronounced hepatic vacuolation coupled with fat cytoplasmic vacuoles, (Fig. 2). Hepatic fatty changes in the group receiving probiotic supplements and HCD were significantly reduced, and there was very slight glycogen infiltration, as shown in Fig. 3. Quantitative scoring of histological sections involving the criteria of the percentage of fat within the hepatocytes showed that the control group scored at grade 0 (2 ± 0.58), HCD group at grade 2 (50 ± 1.58), and the HCD + probiotic group at grade 1 (20 ± 2.89).

**Figure 4:**
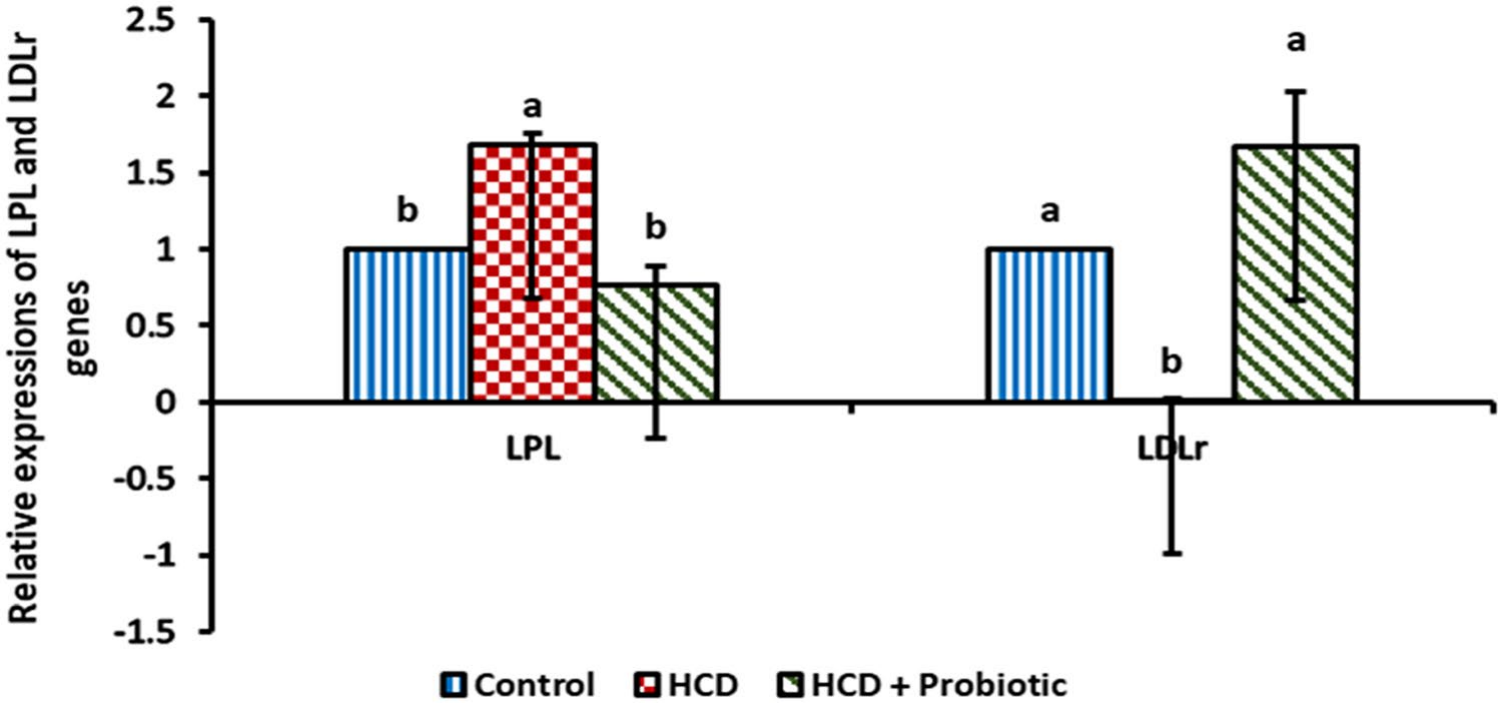
Effect of HCD supplementation and HCD with probiotic supplementation on lipolytic gene expression [Lipoprotein lipase (LPL) and Low-density lipoprotein receptor (LDLr)].

**Figure 5:**
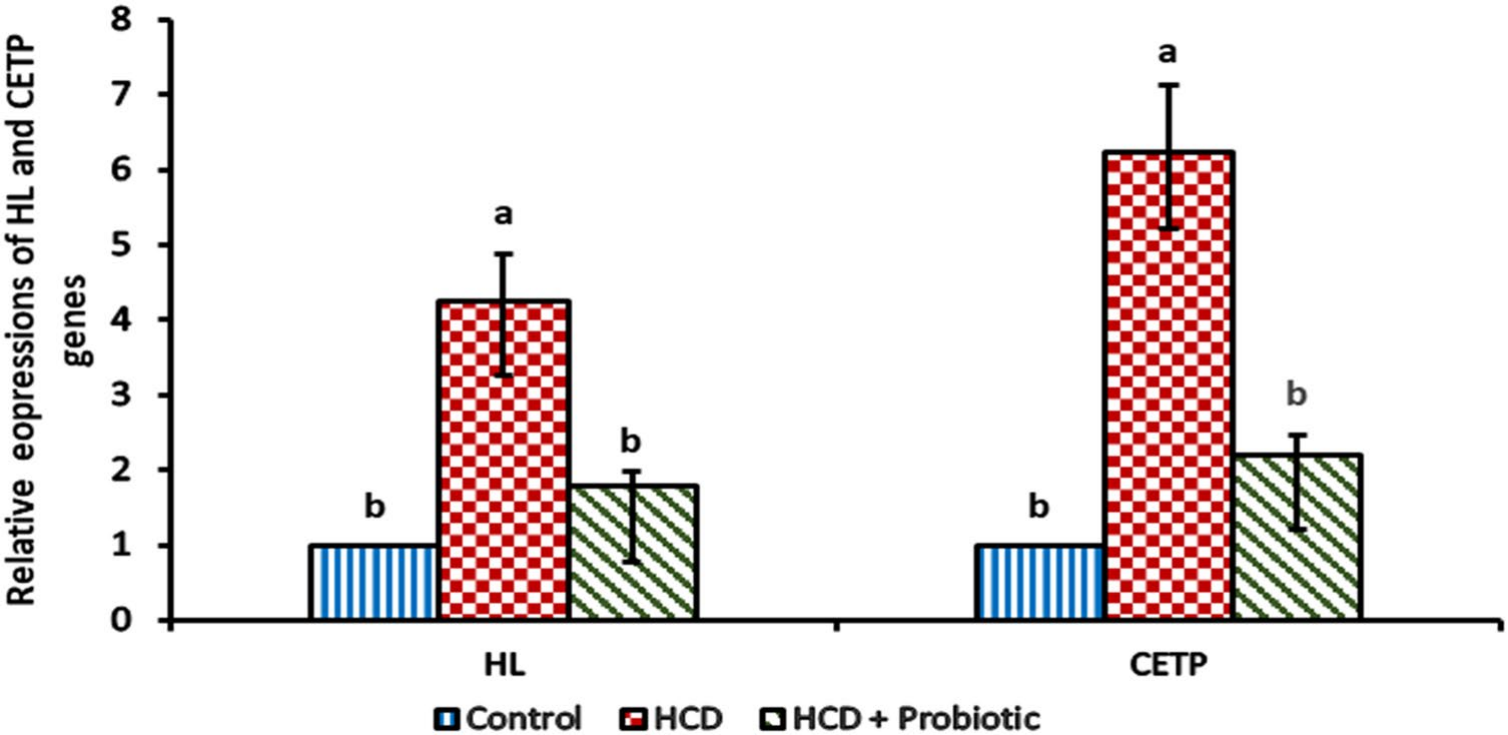
Effect of HCD supplementation and HCD with probiotic supplementation on lipolytic gene expression [Hepatic lipase (HL) and Cholesteryl ester transfer protein (CETP)].

The impact of probiotic supplementation and HCD-induced fatty liver on lipolytic gene expression in the liver was depicted in Figs. 4 and 5. The HCD group showed a significant (*P* ≤ 0.05) increase in *LPL*, *HL*, and *CETP* gene expression as well as a significant decrease in *LDLr* gene expression when compared to the control group and probiotic-supplemented group. Supplementation of probiotics reversed the effects of HCD on gene expression, as no significant differences were found between the control group and probiotic-supplemented group for all genes studied.

**Figure 6:**
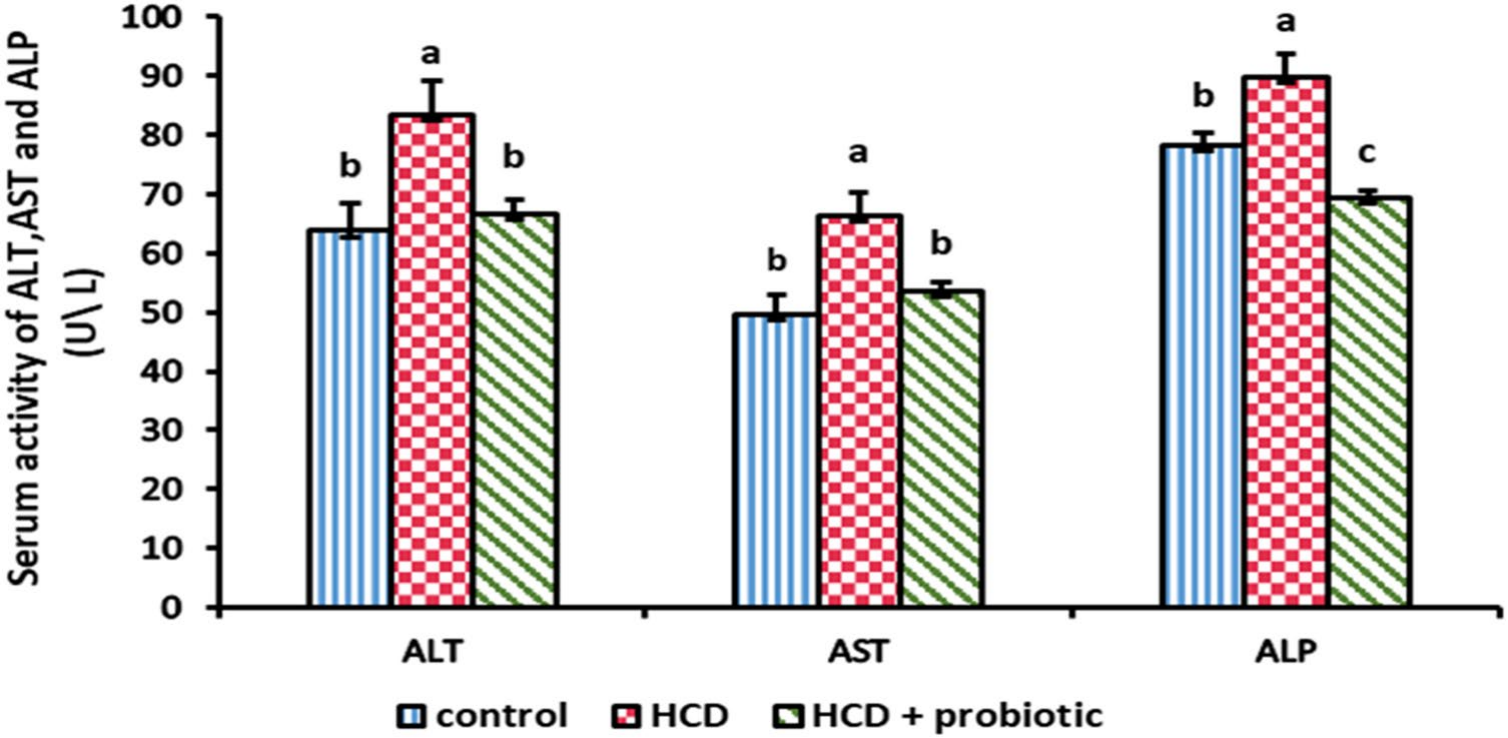
Effect of HCD supplementation and HCD with probiotic on liver enzymes [Alanine Transferase (ALT), Aspartate Transaminase (AST), and Alkaline Phosphatase (ALP)].

**Figure 7:**
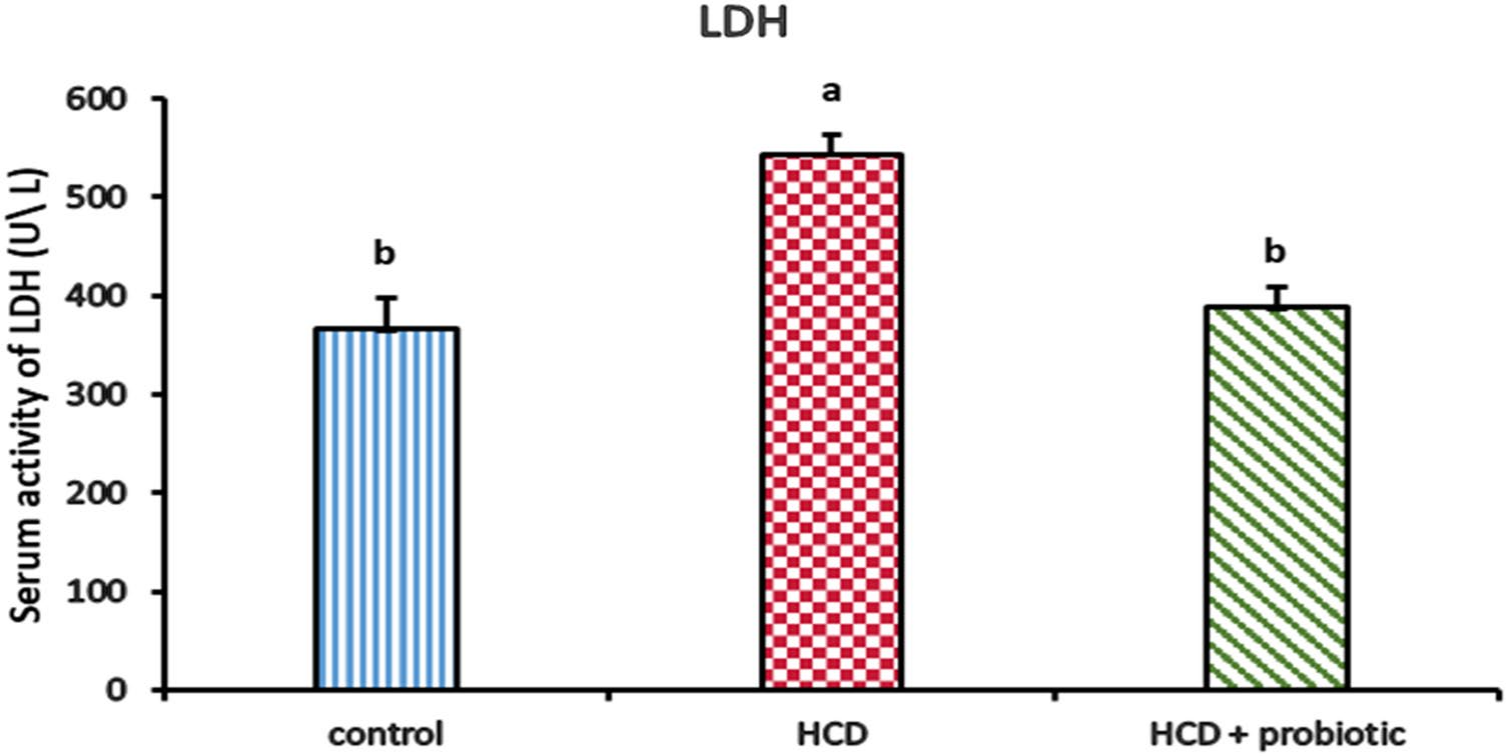
Effect of HCD supplementation and HCD with probiotic on liver enzyme [Lactate dehydrogenase (LDH)].

Figures 6 and 7 indicated the impact of a high-cholesterol diet (HCD) and probiotic supplementation on the liver enzymes ALT, AST, ALP, and LDH. These results showed that the ALT, AST, ALP, and LDH enzymes were significantly higher in the HCD group than in the other groups. There were no significant differences between the control group and the HCD with a probiotic-supplemented group.

**Figure 8:**
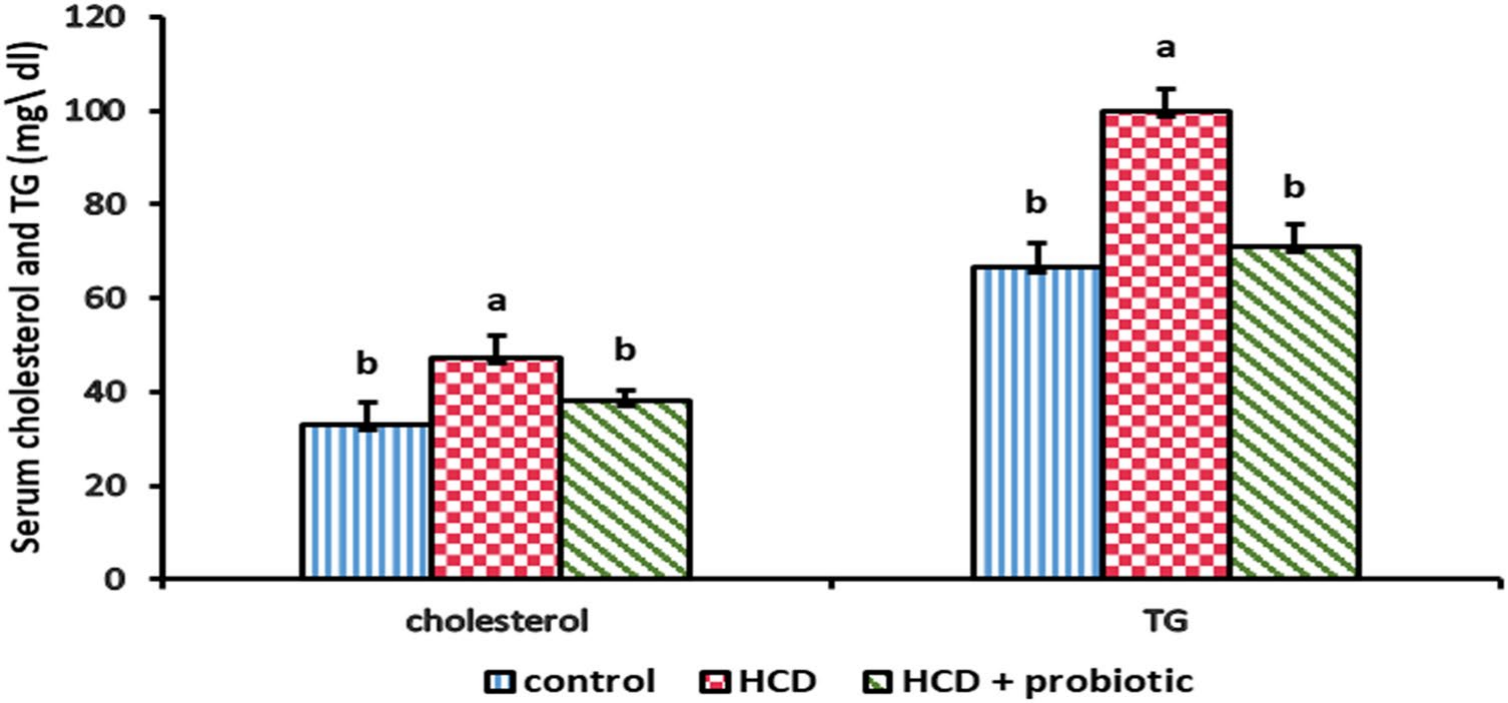
Effect of HCD supplementation and HCD with probiotic on lipid profile [cholesterol and Triglyceride (TG)].

**Figure 9:**
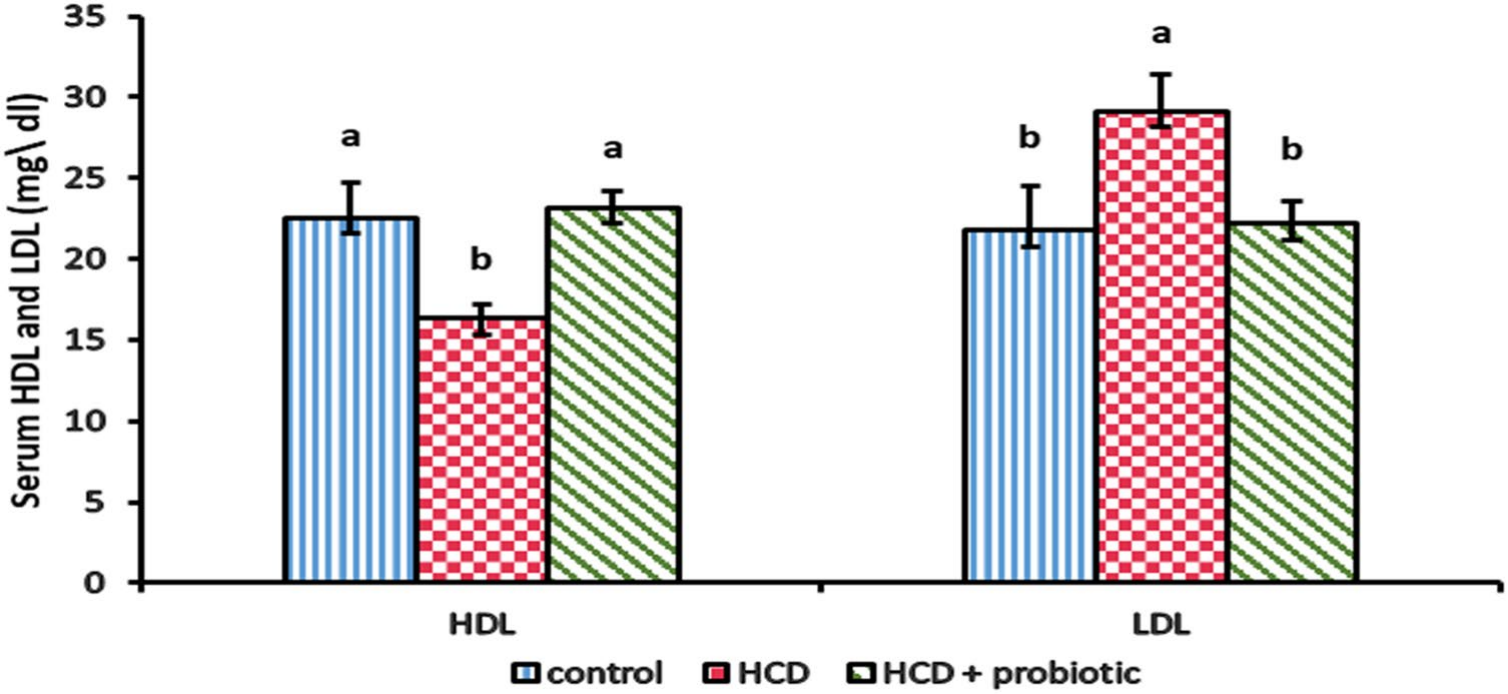
Effect of HCD supplementation and HCD with probiotic on lipid profile [High-Density Lipoprotein (HDL) and low-density lipoprotein (LDL)].

Figures 8 and 9 illustrate how supplementing with probiotics and HCD affects the lipid profile [cholesterol, triglyceride (TG), high-density lipoprotein (HDL), and low-density lipoprotein (LDL)]. These results showed that the HCD group showed a significant increase in cholesterol, TG, and LDL with a significant decrease in HDL compared to the control and probiotic-supplemented groups. The results indicated that probiotic supplementation reversed the effect of HCD.

**Figure 10:**
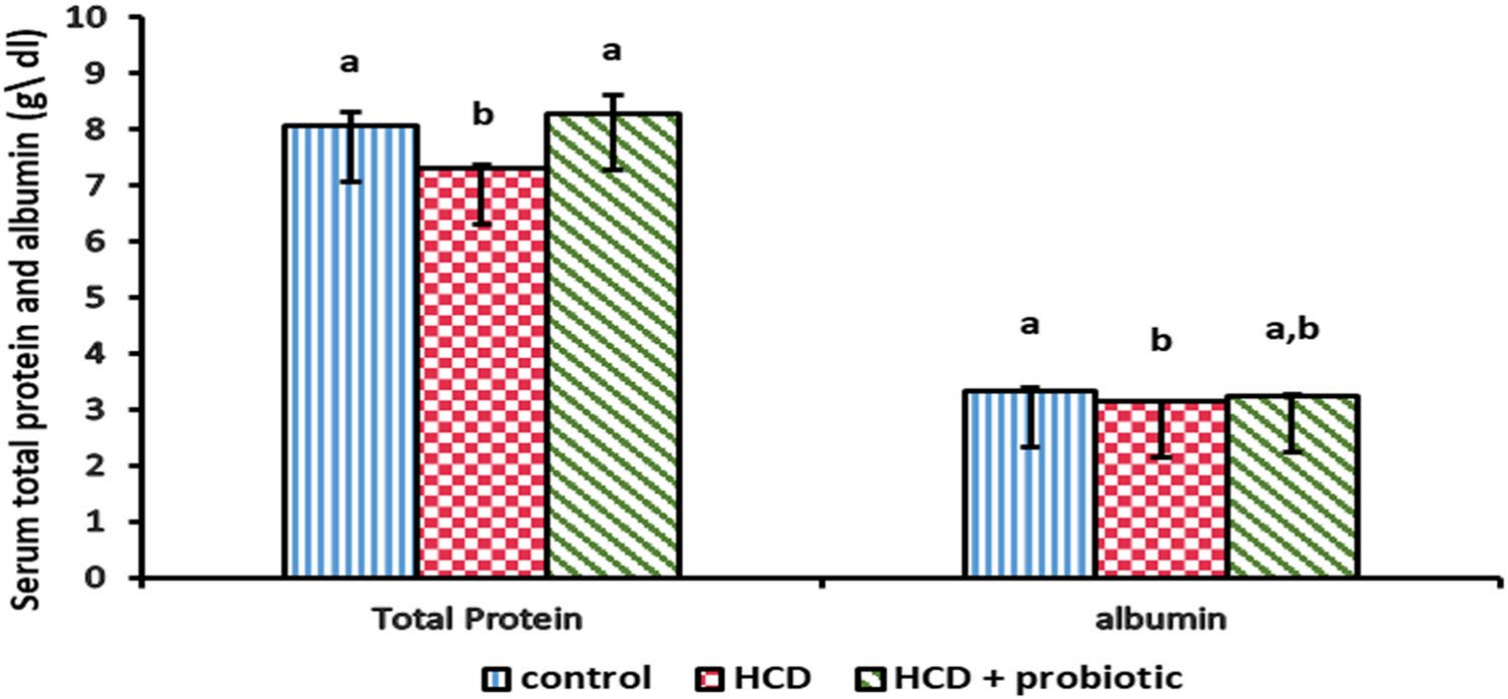
Effect of HCD supplementation and HCD with probiotic on liver function (Total Protein and albumin).

**Figure 11:**
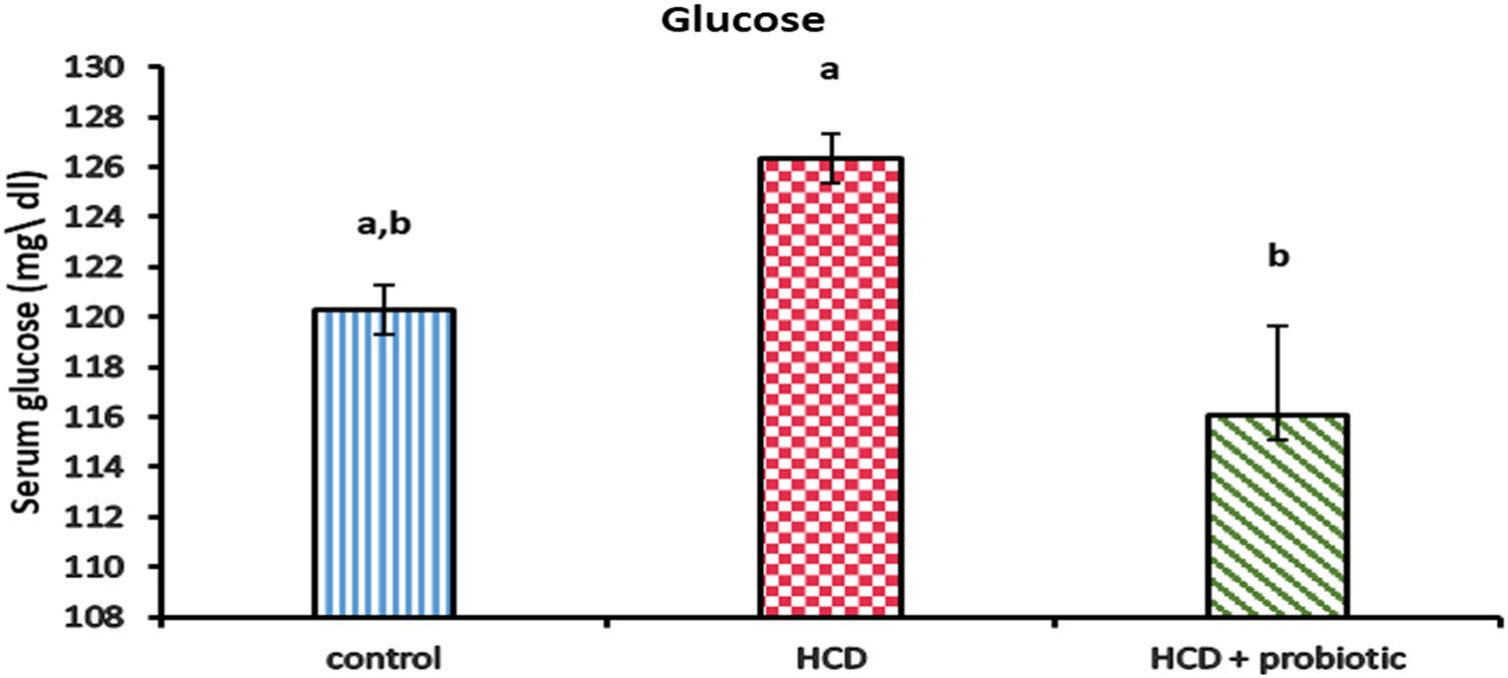
Effect of HCD supplementation and HCD with probiotic on liver function (Glucose).

**Figure 12:**
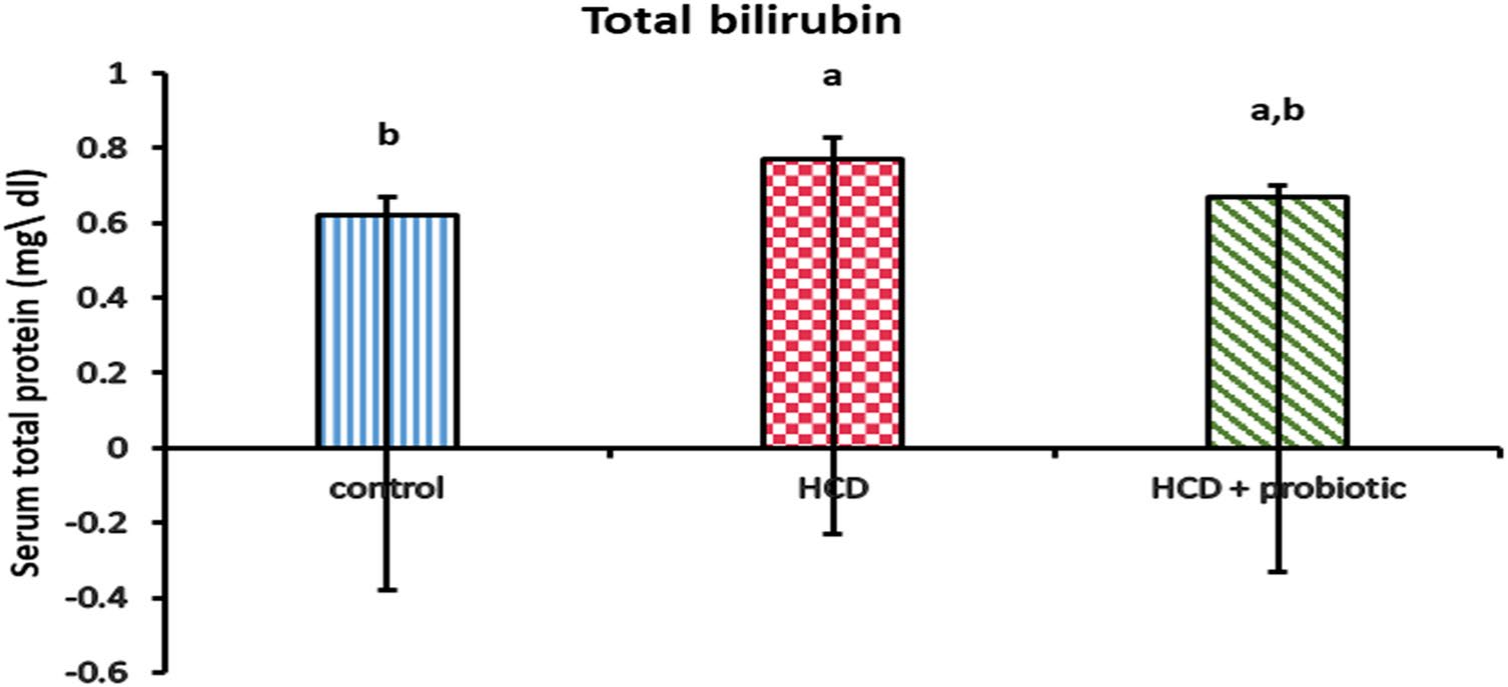
Effect of HCD supplementation and HCD with probiotic on liver function (Total bilirubin).

Figures 10, 11, and 12 showed the impact of HCD and probiotic supplementation on liver function (total protein, albumin, glucose, and total bilirubin). These results demonstrated a marked increase in glucose and total bilirubin, as well as a marked decrease in total protein and albumin in the HCD-supplemented group when compared to the other groups, while the HCD & probiotic-supplemented group exhibited a marked decrease in glucose and total bilirubin, as well as a significant increase in total protein when compared to the HCD group.

**Figure 13:**
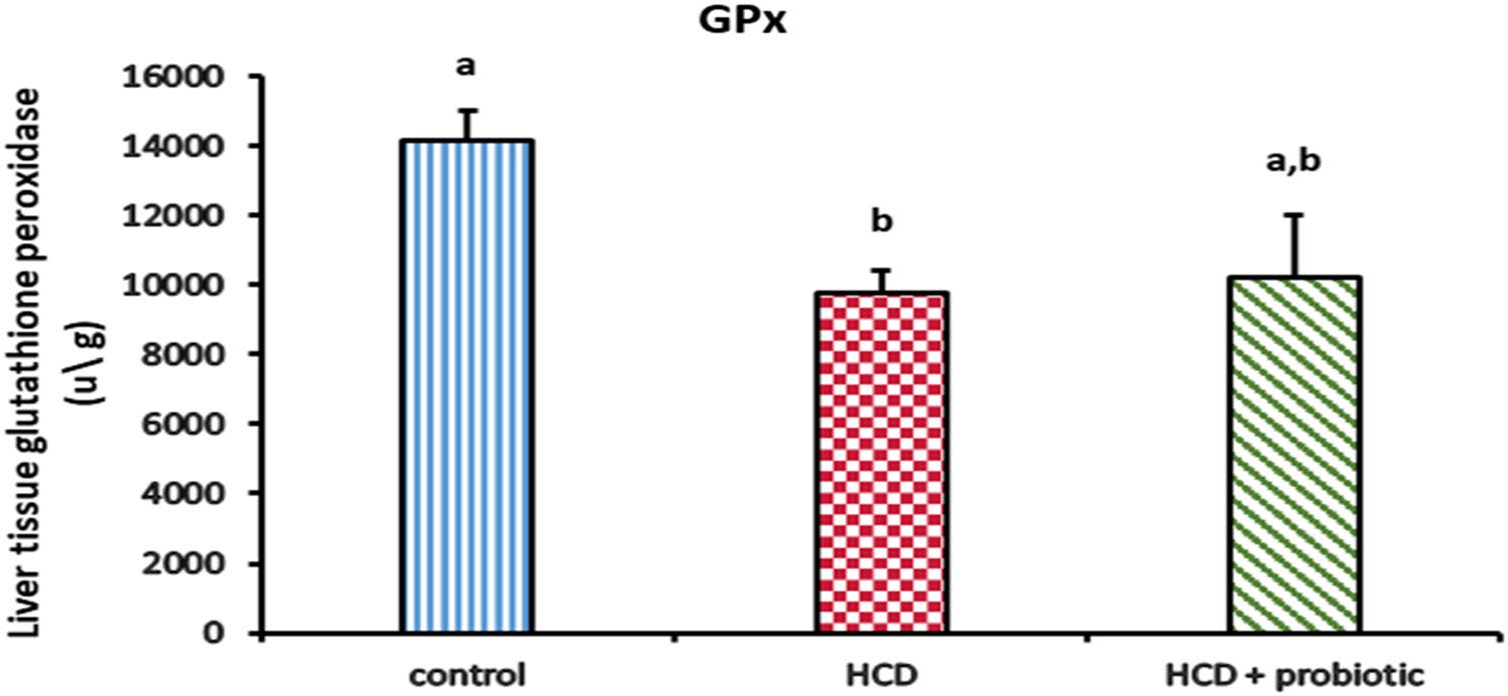
Effect of HCD and HCD with probiotic supplementation on liver antioxidant [Glutathione Peroxidase (GPx)].

**Figure 14:**
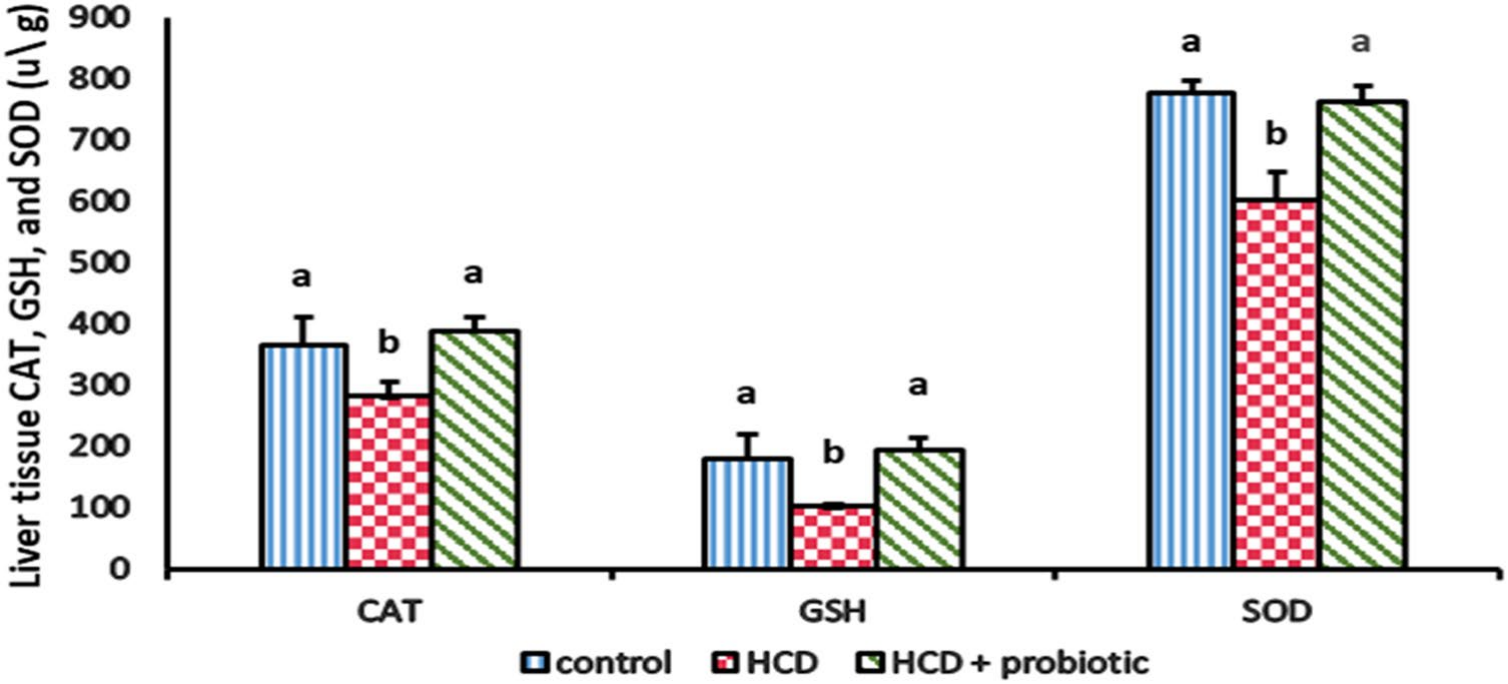
Effect of HCD and HCD with probiotic supplementation on liver antioxidants [Catalase (CAT), Glutathione hydroxylase (GSH) and superoxide dismutase (SOD)].

Figures 13 and 14 showed how probiotic supplementation and HCD-induced fatty liver affect antioxidant levels [glutathione peroxidase (GPx), catalase (CAT), reduced glutation (GSH), and superoxide dismutase (SOD)]. These findings indicate that the liver's concentrations of GPx, CAT, GSH, and SOD were significantly (*P* ≤ 0.05) lower in the HCD-supplemented group than they were in the control group and the probiotic-supplemented group. There were no significant differences in enzyme levels between the probiotic-supplemented group and the control group.

## Discussion

The HCD-supplemented group's liver sections showed significant hepatic vacuolation along with fat cytoplasmic vacuoles. This is similar to Helal et al.^44^, who revealed hepatocytes that appeared to be enlarging and ballooning. In rats with induced fatty liver, cells throughout the hepatic lobule have macrovacuoles scattered throughout the cytoplasm. Also, Wang et al.^45^ demonstrated that there were numerous vacuoles in the hepatocytes of the rabbits fed the HCD, according to an investigation of the liver's histology. Probiotic supplements dramatically minimized hepatic fatty alterations, and there was very little glycogen infiltration. These results are consistent with Rishi et al.^46^, who observed that probiotic administration improved the liver's morphology.

The result showed that there was up-regulation in lipoprotein lipase (LPL) gene expression in the HCD group. This finding is similar to that of Zhang et al.^47^, who discovered that a high-cholesterol diet increases the expression of the LPL gene. Also, similar to Teratani et al.^48^, who reported that NAFLD and nonalcoholic steatohepatitis (NASH) patients' livers had significantly greater levels of LPL messenger RNA (mRNA) expression than did healthy subjects' livers. The development of NAFLD in their mice model is accompanied by a considerable increase in hepatic LPL mRNA levels. Pardina et al.^49^ compared morbidly obese humans with steatosis to control subjects, and LPL mRNA activity was significantly higher (nearly double). Perla et al.^50^ said that increased LPL is linked to NAFLD, while in the HCD plus probiotic-supplemented group, the LPL gene is down regulated. This result is consistent with the findings of Wang et al.^51^, who found that the gene expression of LPL was considerably lower in the *Lactobacillus johnsonii* group than in the control group. Furthermore, Karimi et al.^52^ discovered that probiotic administration, whether single or multiple species, significantly reduced LPL gene expression. LPL expression is increased in both humans and mice by serum obesity-related substances such as leptin, IL-6, and free fatty acids (FFA)^48^. According to reports, the putative signal transducer and activator of transcription 3 (STAT3)-binding site is located in the LPL promoter, and STAT3 signalling elevates LPL expression^53^. It can be claimed that taking a probiotic supplement boosted the expression of (PPARG), which in turn up-regulates the expression of angiopoietin-like 4. (ANGPTL-4). Lower TG levels result from the downregulation of LPL caused by the upregulation of ANGPTL-4^52^.

The LDLr gene expression was lower in the HCD group than in the other groups. This result is similar to that reported by Chen et al.^54^, who reported that, compared to the normal diet group, the high-fat diet group's levels of LDLr mRNA expression were considerably lower. Also, similar to Zhang et al.^47^, who reported that, compared to the control group, the mRNA expression of LDLr was significantly lower in the high-fat, high-cholesterol groups. This is also similar to Xin et al.^55^, who found that evidently, a high-fat, high-sucrose diet reduced the expression of LDLr mRNA and protein in the liver. Also, similar to Song et al.^56^, who reported that, compared to mice on a regular diet, animals on a high-fat diet had significantly fewer LDLr genes in the liver. The expression was higher in the HCD supplemented with probiotics than in the HCD supplemented group. This outcome is comparable to that of Palaniyandi et al.^57^, who discovered that the high-cholesterol diet group supplemented with probiotics had higher levels of LDLr gene expression in the liver than the high-cholesterol diet control group. Tamtaji et al.^58^ discovered that selenium and probiotic-supplemented patients had significantly higher levels of LDLr gene expression than those who just received selenium supplements. Similarly, Song et al.^56^ found that administering *L. acidophilus* NS1 increases LDLr expression in the liver, which was previously suppressed by a high-fat diet.

The expression of the hepatic lipase (HL) gene was increased in the HCD group more than in other groups. This result is similar to that reported by Miksztowicz et al.^59^, who reported that the patients with hepatic steatosis showed considerably higher hepatic lipase activity than controls, and this activity was higher in the most severe state of hepatic steatosis. This result is incompatible with Yang et al.^60^, who reported that hepatic lipase expression levels dramatically dropped in the high-fat diet groups. When compared to the HCD group, the expression of HL in the probiotic-supplemented group was significantly lower. It might be caused by probiotics like *Lactobacillus acidophilus*, which increased HDL and decreased total and LDL cholesterol in experimental animals^61^. Increased hepatic lipase has a role in promoting a more atherogenic profile, as evidenced by the direct correlation between LDL cholesterol and hepatic lipase and the inverse associations with HDL cholesterol^59^.

The expression of cholesteryl ester transfer protein (CETP) was increased in the HCD group. These results are similar to those of Lucero et al.^62^, who reported that patients with hepatic steatosis exhibit elevated CETP activity; they are also similar to those of Lottenberg et al.^63^, who said that a high level of CETP activity is frequently seen in hypercholesterolemic people. Blauw et al.^64^ recorded that it is conceivable that metabolic liver inflammation won't significantly decrease CETP expression and production. The expression of CETP decreased in HCD with the probiotic-supplemented group, as probiotics could have caused it. In test animals, *Lactobacillus acidophilus* boosted HDL and decreased total and LDL cholesterol^61^. It's thought that increased activity of CETP is associated with low HDL^65^.

The obtained data revealed that HCD supplementation in normal rabbits exhibited a significant increase in serum ALT, AST, and ALP activities when compared with the control. These findings are remarkably identical to those of previous investigations^45,66,67^. On the other hand, these results disagree with those of Kainuma et al.^68^, who reported that there is no significant difference between high cholesterol diets and control diets rabbits. The considerable increase in ALT, AST, ALP, and LDH following high-cholesterol diet (HCD)-induced nonalcoholic fatty liver disease (NAFLD) was attributed to an increase in the hepatic cell membrane’s fragility, which caused enzyme release into the bloodstream. Due to the liver's compromised structural integrity, these cytoplasmic enzymes are released into the circulation following an autolytic breakdown or cellular necrosis^69^. The level of these enzymes decreased in HCD with the probiotic-supplemented group. These findings are similar to those of Adesiji et al.^70^ and Li et al.^71^, who reported that *Lactobacillus acidophilus* decreases liver enzyme levels. This might be viewed as a positive side effect of taking *Lactobacillus acidophilus*, which is effective in preserving the health and activity of the epithelial cells lining the biliary duct, showing how probiotics directly affect liver function^72^.

The levels of serum triglycerides (TG) and cholesterol were rising. These outcomes resemble those reported by Sigrist-Flores et al.^73^; Xing et al.^74^; and Lee et al.^75^. TG and cholesterol levels were significantly lower in the HCD probiotic supplemented group compared to the HCD supplemented group. These resemble the reports of Mazloom et al.^76^ and Lee et al.^77^, who reported that *Lactobacillus acidophilus* has a hypocholesterolemic impact and lowers blood triglycerides. Also, Song et al.^56^ reported that hepatic cholesterol and TG levels may be decreased by *L. acidophilus.* Also, Kullisaar et al.^78^ reported that probiotic supplementation dramatically decreased total cholesterol and triglycerides. These results may be attributed to a decrease in the host's intestinal absorption of fatty acids as a result of *L. acidophilus*^79^. Moreover, Park et al.^80^ reported that *L. acidophilus* improves lipid metabolism.

The result showed that there was a decrease in high-density lipoprotein (HDL) and an increase in low-density lipoprotein (LDL) in serum. These findings are similar to those of Paul et al.^81^, who reported that lower blood HDL and greater LDL particle content were independently linked to NAFLD. Also, Briseño-Bass et al.^82^ reported that a rise in LDL and a reduction in HDL were both substantially correlated with hepatic steatosis, and Sigrist-Flores et al.^73^ reported that chronic intake of the fat-enriched diet inducing fatty liver caused a high level of LDL and a low level of HDL. Moreover, Kainuma et al.^68^ reported that many physiopathological characteristics of NAFLD are shared by cholesterol-fed rabbits, where this model may be useful for elucidating the mechanism of NAFLD related primarily to hyperlipidemia because it did not exhibit insulin resistance or obesity. When compared to the HCD-supplemented group, the HCD with probiotic supplementation significantly increased HDL and decreased LDL. These findings are similar to those of Song et al.^56^, who reported that high LDL cholesterol levels may be reduced as a result of *L. acidophilus*-induced liver low-density lipoprotein receptor (LDLr) recovery, which may make it easier for the liver to absorb plasma LDL. Also, Jouybari et al.^61^ found that ingestion of yoghurt containing *Lactobacillus acidophilus* in their experimental animals resulted in a rise in HDL and a decrease in total and LDL cholesterol. Kullisaar et al.^78^ found that due to probiotic use, LDL cholesterol levels and total cholesterol all reduced dramatically, while HDL cholesterol showed a trend to improve. Probiotics are thought to decrease cholesterol by blocking the reabsorption and subsequent excretion of bile salts through their action of deconjugating bile salt, which prevents its recycling^83^.

The findings showed that there was a significant decrease in total protein and albumin in the HCD group compared to other groups. These findings are consistent with those of Helal et al.^44^, who found that fatty liver had a significant decrease in total protein and albumin levels. In addition, Mikolasevic et al.^84^; Grgurevic et al.^85^; and Kawaguchi et al.^86^ reported that patients who had NAFLD showed a low level of serum albumin. The results disagreed with Cho et al.^87^, who reported that patients with fatty liver showed a higher level of total protein. Serum total protein and albumin significantly increased in the HCD group with probiotic supplementation compared to the HCD group. These findings are similar to those of Ayyat et al.^88^, who claimed that taking a probiotic supplement increased serum total protein and albumin levels significantly. Moreover, *Adriani *et al*.*^89^ stated that broiler chicken treated with dry probiotics had the greatest levels of blood protein and albumin. These findings didn't agree with Alkhalf et al.^90^, who declared that their study's probiotic supplementation had no effect on the serum concentrations of total protein or albumin. The inclusion of probiotics in the diet increases the amount of aminoethyl cysteine and lysine analogues in the digestive system, which are then converted to lysine and cysteine amino acids to enhance the retention of proteins important for the development of meat^91^.

The research showed that the HCD group's serum glucose rose in comparison to the other groups. This is analogous to Helal et al.^44^, who reported that fatty liver-induced lab animals had higher serum glucose levels, and likewise in line with Cho et al.^87^ and Paul et al.^81^, who reported that individuals with fatty livers have high serum glucose levels. The results showed that the HCD in the probiotic-supplemented group had a significantly lower serum glucose level. These results concur with those of Adesiji et al.^70^, who noted that the *lactobacillus acidophilus*-treated rat groups showed a considerable reduction in blood glucose levels. This observation can be the result of appropriate insulin release, which helps to control blood glucose levels^92^. Endogenous insulin production may be enhanced by promoting glucose storage in the liver, increasing the body's usage of glucose, or giving probiotics that may have improved the beta cells' declining activity^93^.

The study revealed that the HCD group's serum total bilirubin was significantly higher than that of the other groups. This is similar to Jain and Singhai^94^, who reported that the affected liver's serum bilirubin levels have significantly increased. Nevertheless, HCD with probiotic supplementation significantly reduced serum total bilirubin levels. This agrees with Mutlu et al.^95^, who reported that the probiotic supplementation group in their study had reduced levels of bilirubin. In this case, glucuronidase activity may be inhibited.

The results showed that there was a decrease in liver antioxidants (GPx, CAT, GSH, and SOD) in the HCD-supplemented group. These data are analogues to Videla et al.^96^, who reported that GPx, GSH, CAT, and SOD activity were decreased in NAFLD patients. Compared to the HCD group, these antioxidants were significantly higher in the group receiving probiotic supplements with HCD. These results are consistent with those of Amdekar and Singh^97^, who found that *L. acidophilus* maintained oxidative stress markers in collagen-induced arthritic rats. Moreover, Dowarah et al.^98^ discovered that certain lactic acid bacteria strains might boost the production of antioxidant enzymes or control and alleviate circulatory oxidative stress to shield cells from oxidative stress-related harm. Although the Food and Drug Administration has not approved any medications to treat NAFLD, current treatment options depend on lifestyle modification and dietary restrictions. In addition, treatment is based on the use of antioxidants as probiotics or the treatment of associated metabolic diseases like obesity, type 2 diabetes, and dyslipidemia, which are all directly related to NAFLD. Recently, there are many drugs in the pipeline that are reckoned as good candidates to cure NAFLD/NASH^99^.

## Conclusion

Many of the physiopathological characteristics of NAFLD were shared by cholesterol-fed rabbits. This model may be useful for elucidating the mechanism of NAFLD related primarily to hyperlipidemia because it did not exhibit insulin resistance or obesity. However, the current study showed that the enzymatic activity of the serum liver profile, liver function tests, liver tissue antioxidants and peroxide, and lipid profile were all improved when a probiotic was administered throughout the rabbit's rearing period. Also, the supplements improved the pathological organ damage brought on by HCD.

## Data Availability

The datasets used and/or analysed during the current study available from the corresponding author on reasonable request.
